# I'll Txt U if I Have a Problem: How the Société Canadienne du Cancer in Quebec Applied Behavior-Change Theory, Data Mining and Agile Software Development to Help Young Adults Quit Smoking

**DOI:** 10.1371/journal.pone.0091832

**Published:** 2014-03-19

**Authors:** Trevor van Mierlo, Rachel Fournier, Anathalie Jean-Charles, Jacinthe Hovington, Isabelle Ethier, Peter Selby

**Affiliations:** 1 Evolution Health Systems Inc., Toronto, Ontario, Canada; 2 Société Canadienne du Cancer, Montreal, Quebec, Canada; 3 Centre for Addiction and Mental Health (CAMH), Toronto, Ontario, Canada; Inserm & Universite Pierre et Marie Curie, France

## Abstract

**Introduction:**

For many organizations, limited budgets and phased funding restrict the development of digital health tools. This problem is often exacerbated by the ever-increasing sophistication of technology and costs related to programming and maintenance. Traditional development methods tend to be costly and inflexible and not client centered. The purpose of this study is to analyze the use of Agile software development and outcomes of a three-phase mHealth program designed to help young adult Quebecers quit smoking.

**Methods:**

In Phase I, literature reviews, focus groups, interviews, and behavior change theory were used in the adaption and re-launch of an existing evidence-based mHealth platform. Based on analysis of user comments and utilization data from Phase I, the second phase expanded the service to allow participants to live text-chat with counselors. Phase II evaluation led to the third and current phase, in which algorithms were introduced to target pregnant smokers, substance users, students, full-time workers, those affected by mood disorders and chronic disease.

**Results:**

Data collected throughout the three phases indicate that the incremental evolution of the intervention has led to increasing numbers of smokers being enrolled while making functional enhancements. In Phase I (240 days) 182 smokers registered with the service. 51% (n = 94) were male and 61.5% (n = 112) were between the ages of 18–24. In Phase II (300 days), 994 smokers registered with the service. 51% (n = 508) were male and 41% (n = 403) were between the ages of 18–24. At 174 days to date 873 smokers have registered in the third phase. 44% (n = 388) were male and 24% (n = 212) were between the ages of 18–24.

**Conclusions:**

Emerging technologies in behavioral science show potential, but do not have defined best practices for application development. In phased-based projects with limited funding, Agile appears to be a viable approach to building and expanding digital tools.

## Introduction

Delivering interventions, improving patient safety, reducing cost, and accurately accessing and updating patient-centric information are the major driving forces behind the development of mHealth [1]–[3]. Traditional approaches to digital project development rely on rigid methodologies that can be costly. These rigid methodologies are often problematic as they do not allow for fast-paced evolution, which is required to keep digital programs relevant and usable.

Despite the benefits and promise of mHealth there are significant costs in building and maintaining systems. Costs are generally divided into three types: direct (hardware), indirect (administrative and training) and intangible (the maintenance of software quality) [4]. All three can be daunting for smaller organizations or researchers with limited funding, particularly intangible costs, as they are difficult to predict.

For decades experts in technology and digital procurement have recognized the escalation of costs in software creation, maintenance, and reliability. As early as 1973 it was predicted that software maintenance and development costs would eventually outweigh the acquisition of hardware [5]. Out of this concern arose the field of software engineering economics, which addresses managerial decisions of software engineers that are specifically related to software life cycles and cost estimation [6].

Traditionally, software development processes have been incremental, sequential, and inflexible. The most common example is the waterfall model, a sequential methodology where development flows downward. Waterfall, popular in large-scale enterprise projects, is largely falling out of favor as it does not allow for incremental change, flexibility, or rapid evolution [7], [8].

The shift toward Agile software development framework is an attempt to solve the problems related to waterfall [9]. Although Agile is a broad term it can be defined as an iterative, incremental, and responsive development methodology that involves collaboration through cross-functional and interdisciplinary teams [10], [11].

From cost projection or traditional project management perspectives, critics of Agile express concern that it is poorly defined, is not well published, and is regarded as more of a philosophy than a precisely defined methodology [12]. Past attempts to empirically evaluate Agile have concluded that it is difficult to map findings to a specific process [13]. However, as illustrated through the phased development of SMAT, Agile can be used to work within fixed budgets allocated over particular time frames. This iterative approach allows for real-time adaptive innovatitions that would not be possible in linear or sequential development models.

### Smoking Rates in the Province of Quebec, Canada

Approximately 17.3% of Canadians smoke, with Quebec having the highest percentage of smokers. In fact, smoking prevalence has increased in Quebec from 18.8% in 2010 to 19.8% in 2011 [14]. Smoking rates are higher among young adults in Quebec as over 17% of 15–19-year-olds and over 23% of 20–24-year-olds smoke [15]. Reaching younger smokers, particularly those aged 18–24, is difficult as they traditionally fail to seek treatment [16].

### Digital Smoking Cessation Interventions

Only 3–5% of smokers are able to quit unaided [17]. Smokers require solutions that are tailored, have high reach, and are cost effective [18], [19], and digital health interventions have demonstrated promising results in scientific trials [20].

However, there is very little published research into the feasibility of providing long-lasting, engaging digital mobile health (mHealth) solutions on a population level [21]. Positive results from randomized controlled trials do not report the true costs related to building, scaling, maintaining and innovating widespread services [20].

The purpose of this study is to describe phase-based development and evolution of Service de Messagerie Texte pour Arrêter le Tabac (SMAT: http://www.smat.ca), an mHealth program designed to help young adult Quebecers quit smoking using Agile methodology. SMAT is maintained by the Société canadienne du cancer (SCC) Quebec Division.

## Methods

Anonymous retrospective data were extracted from SMAT's Structured Query Language (SQL) database. All data collection procedures adhered to international privacy guidelines [22]–[24] and were in accordance with the Helsinki Declaration of 1975, as revised in 2008 [25].

As this study was based on unidentifiable archival data, it was deemed exempt from further review. Regulations for the collection, use, and disclosure of personal information set forth by the Office of the Privacy Commissioner of Canada, as outlined in the Personal Information Protection and Electronic Documents Act (PIPEDA), were adhered to. Data from the SMAT website are not publically available. However, researchers interested in reviewing datasets for academic purposes are encouraged to contact Société canadienne du cancer Quebec Division.

**Figure 1:**
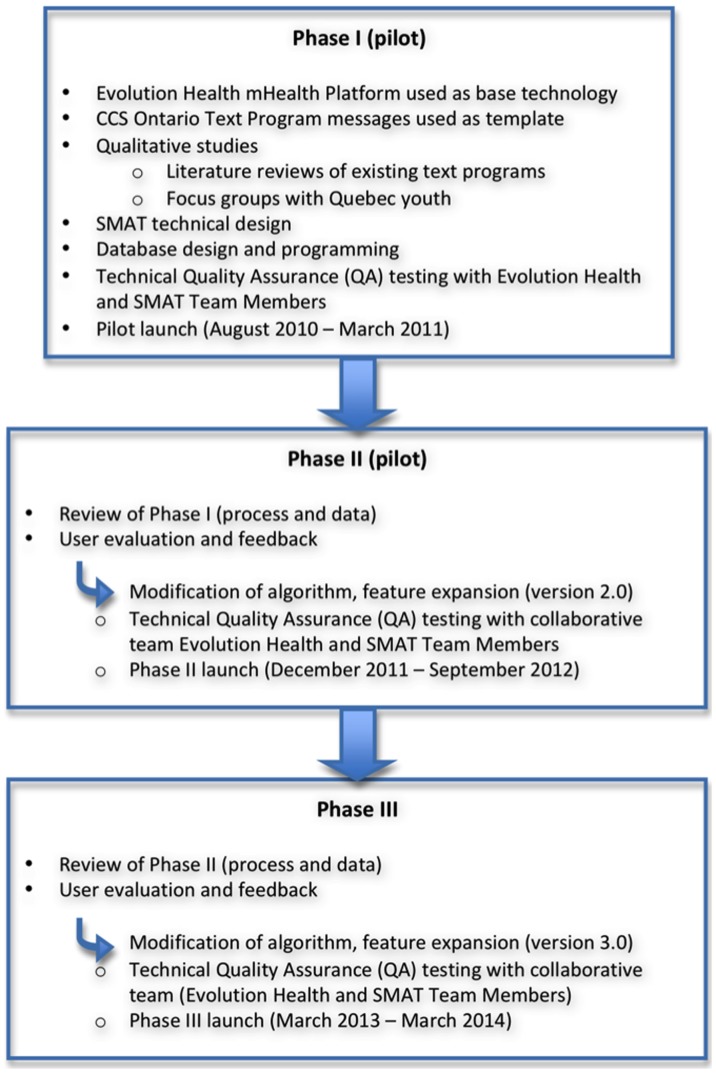
SMAT Development Methodology.

The project was divided into three phases based on the availability of resources per fiscal year. A rough outline for the desired goal and timeline was developed with the funder. A phase-based approach was used to develop and expand SMAT. The original software code and each of the three phases of development followed Agile methodology and involved active input from multiple agencies including behavioral scientists, database architects, software developers, data analysts, marketers, funding agencies, suppliers, project managers and program managers (see Figure 1). The rationale for crowd sourcing these groups was to ensure content, processes, usability, and timelines were realistic and achievable.

### Program Development: Original Source Code and Adoption

The original source code was based on an experimental text-messaging program offered by Evolution Health Systems Inc (EHS). The program was based on the Health Belief Model [26], the Transtheoretical Model [27], and the spirit of Motivational Interviewing [28]. This source code was licensed to the Canadian Cancer Society (CCS) Ontario division, and adapted to English and French as a supplement to the quitline Smokers' Helpline Online (SHO: http://www.SmokersHelpline.ca).

EHS and SHO adapted source code algorithms to appeal to two profiles: current smokers and recent quitters (see Table 1). Reactive text messages were also created to help individuals in urgent situations (keywords: *edgy*, *alcohol*, *stress*, *slip*, *craving*, *smokers*, *motivate*, *stop*).

**Table 1:** SHO Text Messaging Distribution Frequency – Current Smoker and Recent Quitter Profiles.

User Profile	Pre Quit Date	Quit Date	Post Quit Date
Current Smoker	6 days	1 day	13 weeks
Recent Quitter	n/a	n/a	11 weeks

Sample SHO Proactive Text Message:

*As your body adjusts to life without nicotine, edginess is common. Let others know you are not mad at them but just having a hard time.*

*Emergency Coping Plan: AVOID or LEAVE the situation, DISTRACT, DELAY and use positive SELF TALK. Call SHL 1 877 513-5333 for support.*

SHO and EHS researchers collaborated on analyzing message frequency data, and presented findings at the international 2010 mHealth Summit in Washington DC [29].

### Program Development: SMAT Pilot Phase I

In early 2010, Quebec-based SCC sought a novel solution that would engage and support young adults in their quit attempts. In order to make most efficient use of resources, SCC modified the SHO text-messaging platform to suit the unique needs and culture of Quebecers and the young adult target demographic. With a fixed budget and timeline, the project team agreed that the end result would be a text messaging service, but functionality would be determined through the course of development in agreement with Agile development principles. The goal for phase I was to enroll 100 users with 60% in the age range of 18–24.

SCC and EHS engaged in a partnership to facilitate the tailoring process, while conforming to established behavior-change theories. First, a systematic literature review was conducted to establish best practices for delivery of text messaging in healthcare settings. Peer-reviewed literature, grey literature and white papers were included. Search themes includes smoking cessation interventions, young adults' smoking habits, young adults' motivations to quit, and existing text messages for smoking cessation.

Next, tone language and content were tested in four focus groups, two in Montreal and two in Quebec City. Criteria of attendees were age (18–20, 21–24), a desire to quit smoking in the next six months, the use text messaging on a daily basis, and current smoking. Eight participants were randomly selected for each of the four focus groups.

Findings indicated that young adults desired tips and information on physiological changes due to cessation, and that a mature tone should be utilized within messaging. Other findings indicated that the free nature of the program should be promoted, that the program was offered by a provincially recognized not-for-profit was viewed positively, and that the words *help*, *commitment* and *coach* should be avoided. Elements of brief intervention [30] were also implemented into the existing algorithm and message content.

After EHS and SHO collaborated on the design of the new program, a one-week test simulating the service was administered to 12 young adults who were randomly recruited from the general population and wanted to quit smoking. Following the test, 20-minute phone interviews were conducted for feedback. Messages were created in an iterative process, and were based on results from the literature review, focus group testing, simulation testing, and user feedback (see Table 2).

**Table 2:** Sample SMAT Phase I Text Message Selection Process.

Style Tested But Not Chosen (too impersonal and institutional)	Style Chosen (normal language, “cool” but not “too cool”)	Style Tested But Not Chosen (too young, not serious or convincing)
*La plupart des envies disparaissent après qlq mn. Que pouvez-vs faire pour surmonter ou retarder 7 cig? Vs serez heureux de l'avoir fait!*	*Ds 2–3 min., ce sera terminé. En attendant, txt « distraction » ou occupe-toi avec n'importe quoi à portée de main*	*As-tu d fois envie de fumer? Relaaaaax! :P C normal pis ça passe en 2–3 min. Fais qqc en attendant pis ça va partir :)*

After program modification and quality assurance testing Phase I was launched in August of 2010. The pilot program consisted of proactive messages that were strategically delivered according to the user's quit date (see Table 3).

**Table 3:** Phase I Proactive Text Messaging Schedule.

Preparation (One Week)	Quit Day	Week 1	Weeks 2–4	Weeks 5–12
One message per day	Two messages	Two messages per day	One message per day	Two messages per week

In addition to modification of proactive messages, six reactive keywords were implemented into the program (*distraction*, *ennui*, *pause*, *oups*, *party*, *stress*). A library was created to recognize common misspellings and to ask the user to clarify if no alternatives were found.

### Program Development: SMAT Pilot Phase II

**Figure 2:**
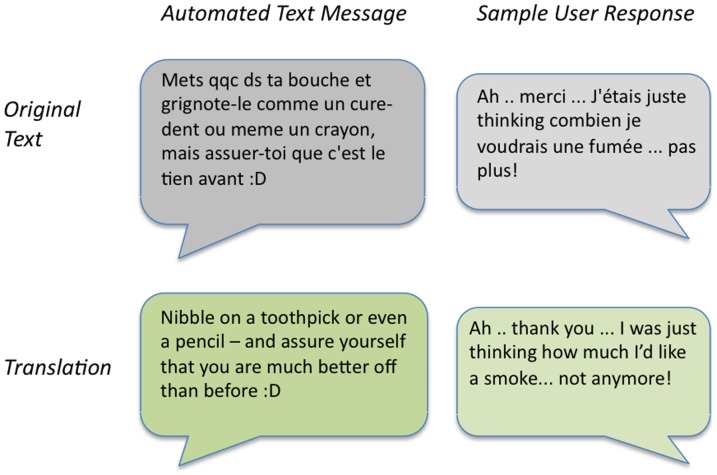
Sample SMAT Phase II conversation with Reactive Text Message keyword *envie* (craving).

Following Phase I, the research teams conducted a detailed review of program statistics, keywords sent to the SMAT service, chat conversations and detailed telephone interviews with those participants who agreed to follow-up (n = 54). Of particular interest in the review of data were conversations that individuals appeared to have with the reactive keyword service (see Figure 2).

Similarly, qualitative feedback from 54 participants indicated that 40% (n = 22) would like to live text chat with a quit specialist. Based on the recurrence of individuals initiating conversations with the reactive service, a text-chat service named Text and Chat Integrated (TaChI) was created.

**Figure 3:**
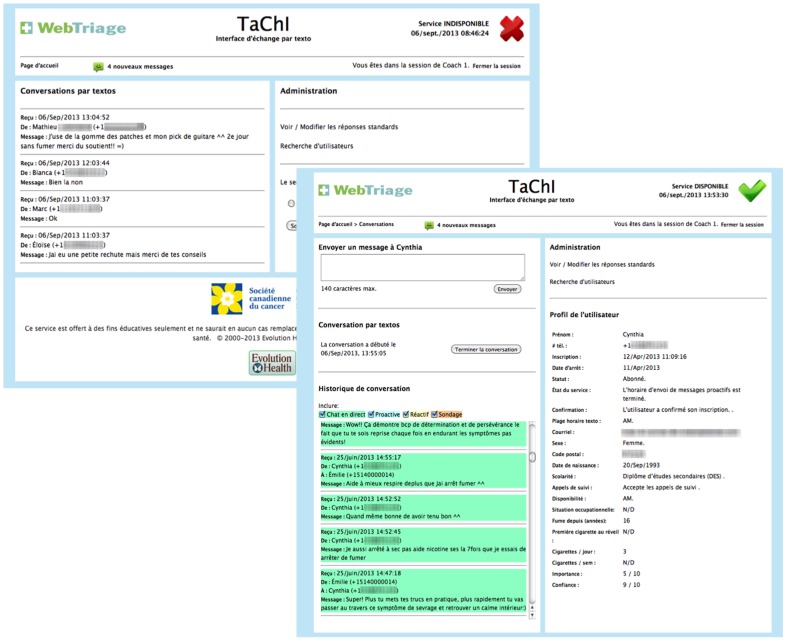
TaChI Interface.

The TaChI web interface allowed telephone quit counselors, from La Ligne j'Arrête, a service also provided by la Société canadienne du cancer who were already part of the existing infrastructure, the ability to engage in real-time text-chats with registered members (see Figure 3). Two specific advantages to TaChI were that the user's demographic profile and chat history would automatically populate the computer screen when they sent a text to a quit specialist providing some context for continuity of care, and quit specialists could chat with multiple registrants at once.

Using Agile methodology, the TaChI interface was co-created by an interdisciplinary team. Informal qualitative interviews and testing processes were held with telephone quit specialists (n = 8) to assess need and functionality, and design interfaces were collaboratively developed with database architects (n = 2) and program managers (n = 3).

Phase II was launched December 6, 2011. Throughout Phase II functional upgrades were made to the TaChI interface based on telephone quit specialist needs, and additional datasets were coded for future analysis. The goal for phase II was to enroll 1,000 users with 60% in the age range of 18–24. Phase II came to a conclusion on March 1, 2012 with text survey data being collected until September 30, 2012.

### Program Development: SMAT Phase III

Phase III was launched March 15, 2013. Data analysis, usage patterns, and input from users and quit specialists were used to enhance SMAT algorithms. Using Agile as a strategy, major upgrades included the ability for users to change quit dates, stop and restart the service, and development of a relapse-recovery algorithm.

To improve personalization, registrants had the option to select tailored algorithms for one or more of six specific conditions: pregnant smokers, those who use other substances such as cannabis, alcohol, or coffee, quitters with mood disorders, students, full-time workers, and those with a chronic disease. Based on their selections the system algorithm delivered tailored messages.

Two additional reactive keywords were added to the service (*ennui* and *difficile*) and one keyword was removed due to its limited use (*voyage*). The program was extended from 12 weeks to 24.

Enhancements to the TaChI interface included the opening and highlighting of a new browser tab when a new text message is received from a user, as well as other functionality upgrades based on quit specialist feedback.

The objective for Phase III was to recruit 1,500 users with 50% under 35 years of age, with a focus on 18–24-year-olds. In addition, SMAT was implemented as an ongoing service rather than a pilot with a focus on evaluation of program sustainability.

## Results

The use of Agile as a methodology, and the iterative, incremental and responsive development process of SMAT is illustrated in Table 4.

**Table 4:** SMAT Program Statistics, and Growth in Functionality.

	Phase I	Phase II	Phase III (ongoing)
	August 4, 2010–March 31, 2011	December 6, 2011–September 30, 2012	March 15, 2013–September 4, 2013
**Registrants**	240	1,103	1,101
**Confirmed Registrants** *	182	994	873
**Proactive messages**	6,108	42,613	44,386
**Reactive keywords**	6	11	12
**Keyword users**	59% (n = 159)	65% (n = 646)	61% (n = 531)
**TaChI chat messages exchanged**		1,099	3,262
**Used TaChI**		38% (n = 374)	60% (n = 523)
**Reset quit date**		8% (n = 83)	13% (n = 115)
**Restarted service**		6% (n = 55)	3% (n = 28)
**Pregnancy algorithm**			1% (n = 11)
**Substance use algorithm**			36% (n = 318)
**Mood disorders algorithm**			19% (n = 163)
**Student algorithm**			10% (n = 85)
**Full-time workers algorithm**			44% (n = 385)
**Chronic disease algorithm**			22% (n = 191)

* Following best practice, SMAT requires a double opt-in.

### Program Evolution

Through the use of Agile, the SMAT program has evolved considerably. Most notable is the high use of TaChI, which was introduced in Phase II and has been used by 38% (n = 374) of registrants in Phase II and 60% (n = 523) in Phase III. Use of TaChI has also intensified; in Phase II (300 days) there were 1,099 messages exchanged, compared to 3,262 so far in Phase III (174 days). The use of TaChI, and the fact that registrants can receive instant feedback when they need it most, reflects past research indicating immediate responses from digital health tools assist in relapse prevention [31], [32].

A further indication of instant support is the fairly consistent use of reactive keywords by registrants (Phase I: 59%, Phase II: 65%, Phase III: 61%). This may also be an indication of SMAT being utilized for relapse-prevention purposes.

Preliminary Phase III results indicate many participants fall into at least one of the six tailored profiles. For example, 44% (n = 385) voluntarily registered for the full-time worker program, and 36% (n = 318) voluntarily registered to receive messages about quitting and substance use. While only 1% of registrants (n = 11) were included in the pregnant smokers algorithm, the percentage reflects the population pregnancy frequency, and this may be encouraging as pregnant smokers are often stigmatized.

In Phase II, 8% (n = 83) reset their quit date. There was an increase in the use of this program enhancement in Phase III, with 13% (n = 115) of registrants using this tool.

Similar to resetting a quit date 6% (n = 55) of users in Phase II restarted the service from where they had left off (if they had stopped the program). Half as many users, 3% (n = 28), have used this feature to date in Phase III.

### Usage Patterns

Considering high rates of smoking among 18–24-year-olds in Quebec, it is encouraging that SMAT is attracting users within this age group. However, usage has been somewhat inconsistent with 62% (n = 112) of registrants being between the ages of 18–24 in the pilot phase, 41% (n = 403) in Phase II, and 24% (n = 212) to date in Phase III (see Table 5).

**Table 5:** Demographic Characteristics.

	Phase I	Phase II	Phase III (ongoing)
	August 4, 2010–March 31, 2011	December 6, 2011–September 30, 2012	March 15, 2013–September 4, 2013
**Number of Days**	240	300	174
**Confirmed Registrants**	182	994	873
**Percentage Male**	51% (n = 94)	51% (n = 508)	44% (n = 388)
**Ages 18–24**	62% (n = 112)	41% (n = 403)	24% (n = 212)
**Average Cigarettes Per Day**	17	17	20

The use of the program by both males and females is also encouraging, with 51% (n = 94) of registrants being male in the pilot phase, 51% (n = 508) in Phase II, and 44% (n = 388) in Phase III.

Also fairly consistent is the average number of cigarettes per day. The pilot phase and Phase II attracted users who smoked an average number of 17 cigarettes per day, while Phase III registrants smoked 20 cigarettes per day.

### Follow-up Survey Results and Quit Rates

At the conclusion of Phase I and II, follow-up surveys were conducted via telephone interviews, web and text surveys.

Following phase I, 28% (n = 74) of registrants were surveyed by web and telephone. 91% (n = 67) reported that they would “definitely” or “probably” recommend SMAT to a friend who was quitting smoking, 86% (n = 64) were satisfied with the service, 73% (n = 54) believed SMAT was useful in their quit attempt, and 57% (n = 42) would have liked SMAT to continue for longer than 12 weeks.

Of survey participants, 43% (n = 32) indicated they had not smoked in the past seven days, resulting in an intent-to-treat quit rate of 12%.

Following Phase II, results from qualitative in-depth telephone interviews (n = 249) and web surveys (n = 124) measured satisfaction with the service. Results indicated that 71% (n = 265) wanted to continue to receive text messages, 62% (n = 231) said SMAT was useful in their quit attempts, and 95% (n = 354) would recommend the SMAT service to others.

Also following Phase II, 3-month follow-up surveys were conducted via text messaging. A total of 27% (n = 273) of registrants responded with 29% (n = 76) indicating abstinence since their quit date. Although not a randomized trial the 3-month self-report intent-to-treat quit rate was 7.6%. It is important to note that results from interviews and surveys are observational.

## Discussion

The goal of this study was to assess whether Agile software development could efficiently build and expand an mHealth intervention through separate phases of funding. Results indicate that despite limited budgets and time restrictions, incremental development through Agile expanded the functionality and value of the program.

Of particular interest is the functional evolution TaChI from Phase II to Phase III. The creation of TaChI was not envisioned at project onset and its development is a result of Agile. Had a traditional methodology to the program development been employed such as waterfall, where project phases and scope are fixed early on, it is unlikely that the TaChI would have been developed.

The modification of reactive messages based on user need increased service usage. At program onset, there were six reactive keywords. In Phase III 12 keywords are now offered. Qualitative analysis of keyword use, misspellings, and common subjects in text chats can improve the functionality and scope of this popular component.

The Phase III development of algorithms targeting six specific conditions is also popular. Agile can also be applied to the development of this type of software component, with the intent of having targeted messages reach special populations with specific needs.

While working within limited budgets and timeframes, Agile can be used in the strategic development of a highly utilized mHealth program that is used by young Quebecers, a group that traditionally fails to seek treatment.

In the Phase I, use amongst young adults aged 18–24 was 62% (n = 112). This decreased to 41% (n = 403) in Phase II, and then decreased 24% (n = 212) in Phase III. While it may be explained through external factors such as seasonality or changes in promotional strategies, age variance should be investigated following Phase III.

From a funding perspective, the ongoing iterative process involved with Agile may be identified as a business risk [33], especially as software development is commonly subject to cost overruns and production delays [34]–[36]. Those working in finance or accounting are responsible for assessing budgets, profitability, reporting and analysis. Chief Financial Officers are well versed in assessing investments with Net Present Value (NPV) or Internal Rate of Return (IRR) analyses, which are metrics that measure a project's profitability in respect to time-specific outgoing and incoming cash flows. Traditionally, software development is formulated with metrics that view projects that are structured in sequences of stages [2], [37]. Financial frameworks and models do exist for Agile, however they need to be commonly embraced if Agile is to become more widely integrated into business strategy [38].

If Agile methodology is continued to be followed beyond Phase III, the project will continue to seek input from behavioral scientists, statisticians, program managers, database designers, project managers and quit specialists. This use of interdisciplinary experts can continue to help assess which SMAT elements are most relevant to the project's objectives, and which elements should be enhanced, added or removed.

In summary, the present study suggests that Agile is a feasible strategy that can transform small-scale digital health projects into full-scale programs with enhanced functionality. Additional studies may help smaller organizations understand how to maximize budgets though interdisciplinary methodologies like Agile.

## Conclusions

Emerging digital health technologies are promising, but do not have defined best-practice application to behavioral science. Due to its iterative nature and relationship to experimental design, Agile is a viable approach for cross-functional teams.

Adopting Agile requires modifications to traditional approaches. As a methodology that lacks an incremental, sequential process it may not be possible to utilize traditional project management software to illustrate predictive schedules. Program improvements are iterative and until they have been fully tested in a user population, they can be subject to errors. To overcome these issues a high degree of communication and cooperation between team members is required.

The primary challenge the SMAT team overcame was to utilize Agile within fixed and limited budgets, and distinct phase timelines. Technical enhancements and future phases will become increasingly complex. For example, the next incremental phase could involve transforming SMAT into both an SMS and mobile application (app). An app would allow for integration with social networking, which could further support relapse prevention. However, a significant cost challenge would be adapting the source code for compatibility with multiple mobile operating systems.

Further work is required in healthcare outcomes economic research that establishes development and maintenance strategies for software designed to have an ongoing population effect.
